# Multiple Primaries: Differences in Survival of Patients with Glioma with or Without Second Malignancies

**DOI:** 10.3390/cancers17213584

**Published:** 2025-11-06

**Authors:** Matthias Demetz, Aleksandrs Krigers, Alexander Miller-Michlits, Adelheid Wöhrer, Claudius Thomé, Christian F. Freyschlag, Johannes Kerschbaumer

**Affiliations:** 1 Department of Neurosurgery, Medical University of Innsbruck, 6020 Innsbruck, Austria; 2 Department of Neuropathology and Neuromolecular Pathology, Medical University of Innsbruck, 6020 Innsbruck, Austria; alexander.miller-michlits@i-med.ac.at (A.M.-M.);

**Keywords:** glioma, multiple primaries, survival, prognostic factors, neuro-oncology

## Abstract

**Simple Summary:**

Gliomas are aggressive brain tumors with limited prognosis, and the influence of a concurrent systemic malignancy on outcome remains unclear. In this retrospective study, we analyzed 426 adult patients who underwent surgery for glioma between 2015 and 2022. Patients were divided into two groups: those with glioma only and those with an additional systemic tumor. A total of 75 patients (17.6%) had a double tumor burden. Patients with a secondary systemic tumor showed significantly shorter overall survival, identifying double tumor burden as an independent prognostic factor for worse outcome. However, patients whose systemic malignancy was in complete remission had similar outcomes to those with glioma only. These findings highlight the clinical relevance of double tumor burden and the need for individualized management and further research into the biological mechanisms behind multiple primary cancers.

**Abstract:**

Background and Objectives: The biological behavior of gliomas is influenced by various factors including molecular features and treatment response. This study investigates the prognostic implications of a second tumor in patients with glioma at time of diagnosis. Given the increasing number of patients presenting with multiple primary malignancies due to improved cancer survival and diagnostic accuracy, understanding the influence of double tumor burden on glioma outcomes is of growing clinical relevance. Methods: We retrospectively analyzed adult patients with intracranial gliomas (WHO grade 2–4), who were surgically treated between 2015 and 2022 at our institution. Patients were categorized into two groups: glioma only and glioma plus additional solid malignancy. We compared progression-free survival (PFS) and overall survival (OS) using Kaplan–Meier and Cox regression analyses. Results: Among 426 glioma patients, 75 (17.6%) harbored a second non-brain tumor. Patients with multiple primaries showed significantly poorer OS (median 6 vs. 14 months, *p* = 0.002). No significant difference in PFS or OS was observed for patients in case the systemic tumor was in complete remission as compared to those with sole glioma. However, patients with progressive or stable systemic tumor had significantly worse outcomes regarding OS (*p* < 0.05). Conclusions: Our findings suggest that the presence of a second systemic malignancy is an independent prognostic factor for worse outcome. Further studies are mandated to elucidate genetic situations and refine therapeutic strategies for these patients.

## 1. Introduction

Gliomas are the most common primary malignant brain tumors in adults and are associated with significant morbidity and mortality [1,2,3,4,5,6]. While diagnostic strategies have improved, prognosis remains poor, particularly in high-grade gliomas [7,8,9]. In recent years, multiple risk factors influencing overall survival (OS) have been identified. These include molecular features such as isocitrate dehydrogenase (IDH) mutation status, surgical parameters like the extent of resection (EOR), socio-economic factors such as higher educational attainment, and the implementation of treatment protocols including the Stupp protocol and, more recently, the use of targeted treatments like Vorasidenib [1,10,11,12,13].

Despite this modest progress in glioma management, the advances in extracranial solid cancers might impact glioma outcomes, because of a rising number of cancer survivors, who may develop gliomas at a later stage. As diagnostic techniques improve and life expectancy increases, the number of patients presenting with more than one tumor is constantly increasing [14,15]. The clinical relevance is growing, yet data evaluating the clinical co-occurrence between gliomas and systemic malignancies are limited.

Despite the growing number of patients affected by both glioma and a preexisting systemic malignancy, there is a notable lack of data addressing how this multiply primary situation influences prognosis and treatment strategies. Patients in this situation face multiple challenges, including limited therapeutic options due to overlapping toxicities, competing prognostic risks, and the burden of navigating two disease trajectories [16,17,18]. For treating physicians, balancing treatment regimens and aligning therapeutic goals becomes increasingly complex, especially when standard protocols do not account for coexisting cancers. Whether the presence of a systemic solid malignancy significantly worsens survival or alters the biological behavior and treatment response of gliomas remains unclear, highlighting the need for further research in this area.

This study aims to investigate the prognostic impact of an additional systemic solid tumor diagnosis in patients with glioma in order to identify the influence on clinical outcome. Identifying any relationship may support more informed prognostic assessments and treatment decisions in this patient population.

## 2. Materials and Methods

This study was conducted using a monocentric retrospective study design. We included all patients aged ≥18 years at the time of surgery who underwent first surgery for an intracranial glioma between 2015 and 2022 at our institution. Patients with recurrent gliomas were excluded from the analysis.

Surgical resection was carried out as the standard treatment approach for all eligible patients. In cases where gliomas were located in non-resectable areas, biopsy was followed by adjuvant radio-chemotherapy [12]. Postoperative follow-up was scheduled at regular intervals, depending on the diagnosis and molecular characteristics. Assessment of tumor progression was performed in accordance with the Response Assessment in Neuro-Oncology (RANO) criteria [19]. All patients, including those with dual tumor burden, were discussed in an interdisciplinary tumor board, where treatment decisions were made individually based on performance status, oncological stage of the systemic malignancy, and patient preference. However, eligibility, initiation, and timing of adjuvant chemoradiation for glioma were determined according to established international treatment guidelines. Individual adaptations were made only when clinically required based on the patient’s overall condition and oncological stage. The general condition of patients was assessed before and after surgery using the Karnofsky Performance Status (KPS) and the Clinical Frailty Scale (CFS). Neuropathological evaluation was routinely performed on formalin-fixed paraffin-embedded (FFPE) tissue. Histopathological diagnoses were established according to the 2021 WHO Classification of Tumors of the Central Nervous System (revised 6th edition) [20]. For patients originally diagnosed based on the 2016 WHO classification, diagnoses were updated to align with the 2021 criteria, wherever possible. IDH1 R132H mutational status was assessed by immunohistochemistry (IHC); in patients under 50 years of age with negative IHC findings, wild-type status of IDH1 and IDH2 was confirmed by DNA sequencing. Nuclear ATRX and EGFR expression, as well as MIB-1 as a proliferation marker, were evaluated by immunohistochemistry (IHC). In cases where IDH, ATRX, or EGFR status had not been previously assessed and sufficient tissue was available, these markers were re-evaluated. The diagnosis of glioma was based on histopathological verification performed by board-certified neuropathologists at our institution in accordance with current WHO classification standards. The diagnosis of the systemic malignancy was based on detailed patient records obtained from other departments, including oncology, internal medicine, and surgery, where these tumors had been initially diagnosed and treated.

In addition to identifying patients with an independent systemic solid tumor diagnosis at the time of glioma diagnosis, medical records were thoroughly reviewed to assess the clinical status of the systemic malignancy. This included the evaluation of available staging scans and oncological follow-up data. Based on these records, the stage of the systemic tumor was categorized according to international oncological standards as complete remission (CR), stable disease (SD), or progressive disease (PD). For subgroup analysis, patients were categorized into two main groups: those diagnosed with glioma only, and those with glioma plus at least one independent systemic solid malignancy. Among patients with a systemic tumor, further stratification was performed based on the oncologic status of the systemic disease.

Statistical analyses were performed using IBM SPSS Statistics (Version 27.0 for Mac OS; IBM Corp., Armonk, NY, USA). Scale variables were assessed for normality and analyzed using *t*-tests when normally distributed, with results presented as mean ± standard deviation (SD). In cases of non-normal distribution, the Mann–Whitney U-test was applied, and results were reported as median with interquartile range (IQR). Categorical variables were compared using the Chi-squared test. Progression-free survival (PFS) and overall survival (OS) were calculated using the Kaplan–Meier method, with differences between groups evaluated using the log-rank test. Cox proportional hazards regression analysis was used to calculate hazard ratios (HRs) and corresponding 95% confidence intervals (CIs) for oncological progression and mortality. The multivariate Cox regression model included all relevant clinical and molecular covariates like age, IDH status, WHO grade, KPS, and extent of resection as well as tumor volume. A *p*-value below 0.05 was considered statistically significant.

Ethical approval was obtained from the Ethics Committee of the Medical University of Innsbruck (1291/2024), and this study was conducted in compliance with the Declaration of Helsinki and its later amendments. Due to the retrospective study design, informed consent was not required according to local ethical guidelines.

## 3. Results

### 3.1. Patient Characteristics

A total of 426 patients were included in the analysis, comprising 56.6% males and 43.4% females, with a median age of 64 years (IQR 53–75). The majority of patients (*n* = 355) were diagnosed with a glioblastoma, IDH-wildtype, CNS WHO grade 4, while 46 patients had an Astrocytoma, IDH mutant, CNS WHO grade 2–4 and 25 patients were diagnosed with an Oligodendroglioma, IDH-mutant, CNS WHO grade 2 or 3. IDH mutation was present in 16.6% of patients, whereas 83.4% had IDH-wildtype glioblastoma. At the time of glioma diagnosis, 75 patients (17.6%) were found to have an additional, independent systemic malignancy. At the time of glioma diagnosis, 45 patients with a solid systemic malignancy were in CR, 11 had SD, and 19 exhibited PD based on their most recent oncological staging prior to glioma diagnosis. Median time from diagnosis of the systemic tumor to diagnosis of the glioma amounted to 49 months (IQR 14–74). Of the 75 patients with a systemic malignancy, 66 patients (88%) had one additional tumor, 7 patients (9%) had two distinct systemic tumors, and 2 patients (3%) presented with three or more additional malignancies. The most frequently observed primary tumors included prostate cancer (12 cases), breast cancer (10 cases), melanoma (8 cases), and non-small cell lung cancer (NSCLC) (7 cases).

**Figure 1:**
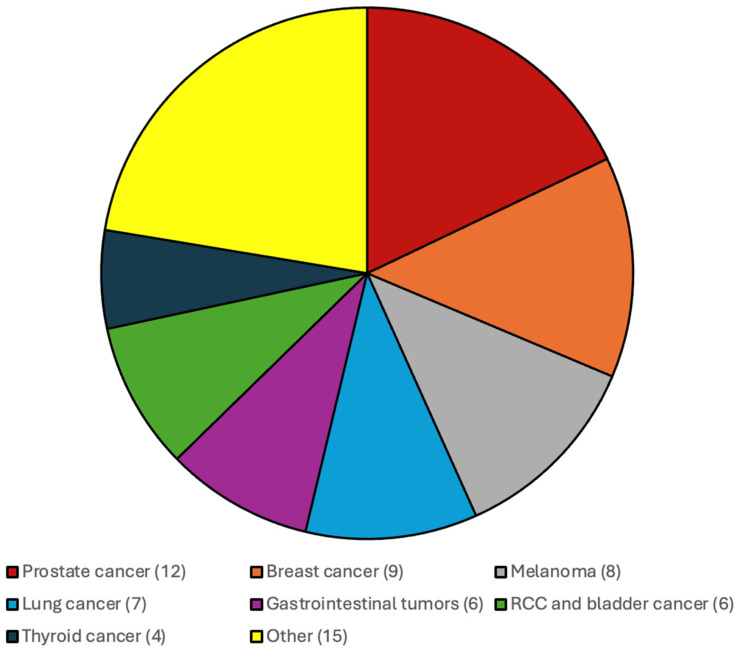
Most frequent additional systemic tumors among patients with CNS WHO grade 4 IDH-wildtype gliomas were prostate cancer, breast cancer, melanoma and gastrointestinal malignancies.

**Figure 2:**
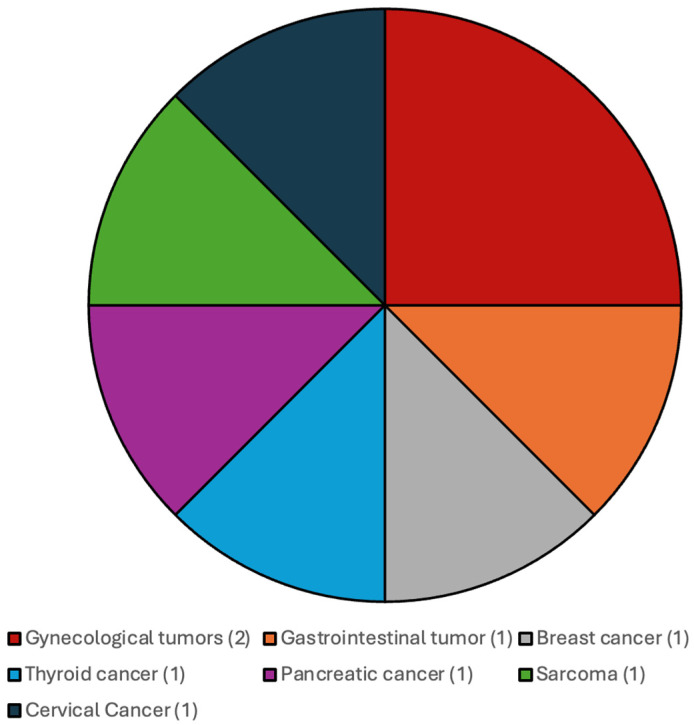
Most frequent additional systemic tumors among patients with CNS WHO grade 2 and 3 gliomas, IDH-mutated.

The distribution of the additional systemic malignancies in patients with CNS WHO grade 4 IDH-wildtype gliomas and IDH-mutated gliomas and CNS WHO grade 2 and 3 gliomas are illustrated in Figure 1 and Figure 2.

Baseline characteristics and comparisons between patients with glioma only and those with an additional systemic tumor are presented in Table 1.

No significant differences were observed between patients with glioma only and those with an additional systemic malignancy in terms of gender distribution, preoperative symptoms, presence of preoperative epilepsy, tumor location, involvement of eloquent brain regions, or surgical treatment modality (all *p* > 0.05).

### 3.2. Histopathological and Molecular Features

Glioma grading significantly differed between the two groups. Among patients with glioma only, 80.9% had CNS WHO grade 4 tumors, 11.6% had grade 3, and 7.5% had grade 2 gliomas. In contrast, patients with multiple primaries showed a markedly higher proportion of grade 4 tumors (93.2%), with only 4.1% presenting grade 3 and 2.7% grade 2 gliomas (*p* = 0.012). Ki-67 proliferation index in glioma tissue was significantly higher in the multiple primary group (*p* = 0.045), and these patients were also significantly more likely to have IDH-wildtype gliomas (*p* = 0.024). Patients without a solid systemic tumor had a significantly higher frequency of advanced EGFR expression (50.9% vs. 38.1%, *p* = 0.042).

### 3.3. Survival and Outcomes

**Figure 3:**
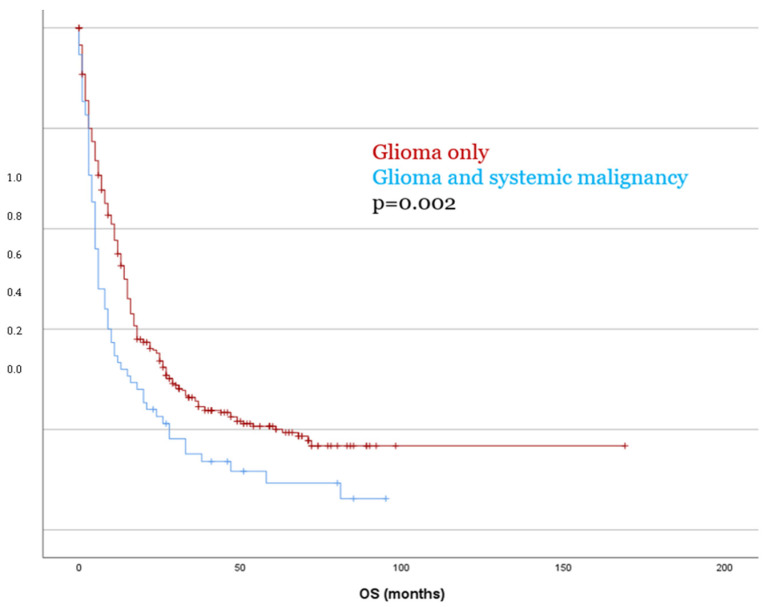
Patients with dual tumor burden showed significantly worse OS compared to patients with glioma only.

Kaplan–Meier survival analysis revealed no significant difference in PFS between patients with glioma only and those with a second tumor diagnosis (*p* = 0.153). In contrast, OS was significantly reduced in patients with dual tumors as compared to those with glioma only, with a mean OS of 18.5 months versus 41.2 months, respectively (*p* = 0.002, Figure 3). These findings were confirmed in the multivariate Cox regression analysis (OS *p* = 0.003), which identified the presence of a systemic malignancy as an independent predictor of poorer overall survival. These findings were consistent in separate analyses for both IDH-mutant and IDH-wildtype glioma (*p* < 0.05).

**Figure 4:**
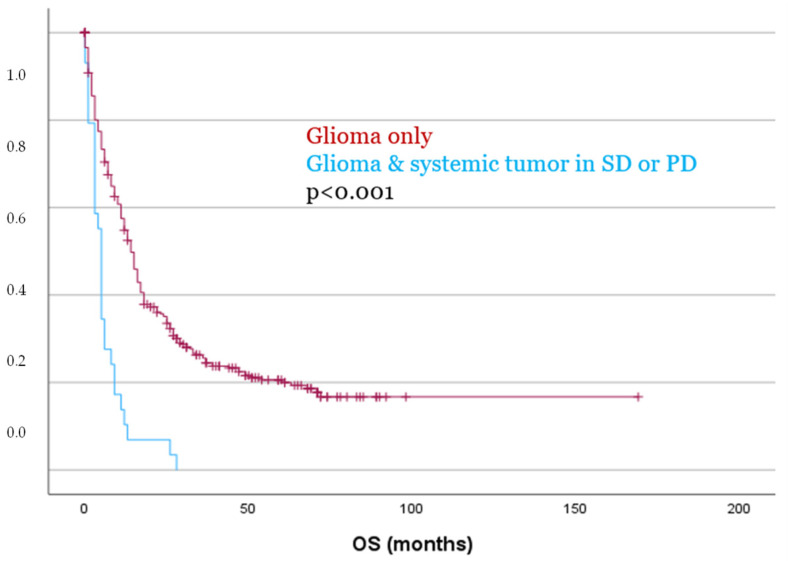
Patients with glioma and a systemic tumor in SD or PD showed significantly worse OS as compared to patients with glioma only.

Even more pronounced differences were observed when comparing patients without a systemic tumor to those with a systemic malignancy in SD or PD at the time of glioma diagnosis. Both PFS and OS were significantly reduced in this group, with a mean PFS of 7.2 months vs. 22.2 months (*p* = 0.002), and a mean OS of 6.3 months vs. 41.2 months (*p* < 0.001, Figure 4), respectively.

Statistically significant differences were also confirmed between patients with CR of their systemic tumor and those with SD or PD. PFS was significantly longer in the CR group (mean 38 vs. 8 months, *p* < 0.001), as was OS (mean 46 vs. 8 months, *p* = 0.035).

**Figure 5:**
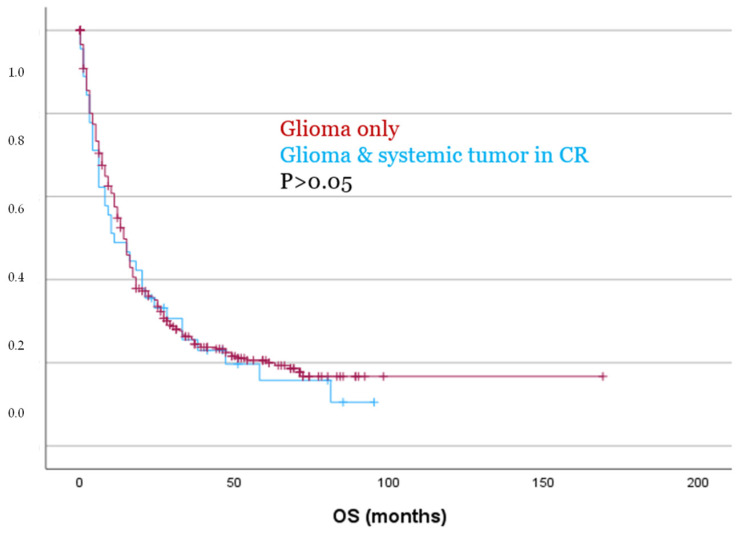
Patients with glioma and a systemic tumor in CR showed no significant differences for OS compared to patients with glioma only.

However, despite being significantly older (*p* = 0.009) and exhibiting significantly higher Ki67 expression levels (*p* = 0.043), patients with a systemic tumor in CR showed no significant differences in PFS or OS as compared to patients with glioma only, as demonstrated by both Kaplan–Meier analysis and Cox regression (*p* > 0.05, Figure 5). These findings were also confirmed in separate analyses for both IDH-mutated and IDH-wildtype gliomas (*p* > 0.05).

No significant differences in PFS or OS were observed between patients with PD and those with SD (*p* > 0.05).

## 4. Discussion

In this study, we identified a prior systemic tumor diagnosis as an independent prognostic factor for worse outcome in glioma patients. To the best of our knowledge, this is the first study to systematically investigate the prognostic impact of a concurrent systemic malignancy in patients with glioma. These findings suggest that multiple primaries may be an underestimated clinical challenge that warrants greater attention in both research and treatment planning. PFS was not significantly different between groups, patients with both glioma and a systemic malignancy had significantly shorter OS as compared to those with glioma only. These effects were particularly pronounced in patients with SD or PD of their systemic tumor. In contrast, patients with a systemic tumor in CR at the time of glioma diagnosis showed no significant difference in PFS or OS. These findings indicate that patients with glioma and a second independent malignancy, particularly those with active systemic disease, may require individualized therapeutic strategies to optimize outcomes. Further studies are warranted to investigate potential genetic aberrations.

Over the past decade, numerous prognostic factors influencing glioma patient outcome have been identified, including molecular markers such as IDH mutation status, the extent of resection, socioeconomic parameters, and evolving treatment modalities like targeted therapies such as Vorasidenib [1,10,11,12,13,21,22,23,24]. However, with the rising age and the incidence of multiple tumors, clinicians are more frequently encountering individuals with both glioma and an additional systemic tumor [15]. While few oncological studies have shown that multiple coexisting primary tumors can negatively influence patient outcomes, this relationship has not been systematically examined in glioma populations [25,26,27]. The prognostic and therapeutic implications of such dual diagnoses remain poorly understood. Therefore, this study aimed to evaluate how the presence and activity of a second systemic malignancy influences survival outcome in glioma patients.

The baseline characteristics in our cohort, including age distribution, gender ratio, tumor grade, and molecular profile, were comparable to those reported in the literature for glioma patients [28,29,30]. Notably, 17.6% of patients presented with a dual tumor burden, a considerable proportion that underscores the clinical relevance of investigating the impact of a concurrent systemic malignancy in this population.

Dual tumor patients showed lower preoperative performance scores, likely due to significantly higher age and a prior cancer history, including exposure to systemic chemotherapy and its associated toxicities [31,32]. These factors may contribute to increased frailty and reduced functional reserve at the time of glioma diagnosis. Moreover, the higher age and WHO grade observed in this cohort may underlie the lower incidence of IDH mutation and the significantly elevated Ki67 index, both of which are known to correlate with more aggressive tumor behavior and poorer prognosis [10,33,34].

We found no significant differences in surgical treatment between the patient groups, with both dual tumor and glioma-only patients receiving similar approaches. Additionally, there was no significant difference in the likelihood of receiving adjuvant therapy between the two groups. However, in cases of PD of the systemic tumor, patients with dual tumor burden were more frequently managed with BSC only after glioma diagnosis. The presence of an additional systemic malignancy likely influenced treatment decisions, with clinicians opting for a more conservative approach due to the patient’s overall prognosis [35,36].

Our findings demonstrate that the presence of a solid systemic tumor at the time of glioma diagnosis was an independent prognostic factor for worse OS. Patients with dual tumor burden had significantly reduced OS compared to those with glioma alone, emphasizing the impact of a concurrent malignancy on survival. This relationship remained robust even after adjusting for other clinical variables, indicating that the presence of a solid systemic tumor independently contributes to poorer outcomes in glioma patients. However, when the systemic cancer was in CR at the time of glioma diagnosis, no significant difference in OS was observed between patients with glioma only and those with dual tumor burden. This suggests that the clinical impact of the second tumor may be less pronounced when it is well-controlled, underscoring the potential for improved outcomes with effective management of the solid systemic malignancy [37,38].

An additional systemic tumor together with the glioma is akin to fighting on two fronts, with the patient facing the challenges of both cancers simultaneously. This dual burden likely limits prognosis, as treatment strategies must be adjusted to account for the competing demands of managing both malignancies [39,40]. Furthermore, the presence of a second malignancy often coincides with other adverse factors, such as increased age and more aggressive molecular features (e.g., higher Ki67 expression, lack of IDH mutation). Together, these factors may compound the negative impact on OS. This complex interplay between the glioma and the additional systemic tumor underscores the importance of considering the dual tumor burden in prognostic assessments and treatment decisions.

Interestingly, patients whose systemic malignancy was in CR at the time of glioma diagnosis were significantly older and exhibited higher Ki-67 indices, yet their survival outcomes did not differ from those with glioma only. This observation suggests that age and proliferative activity alone cannot fully explain the poorer prognosis observed in patients with dual tumor burden. Rather, it indicates that the activity of the systemic malignancy, reflected by stable or progressive disease, likely plays a decisive role in influencing overall survival.

Several mechanisms may contribute to the worse OS observed in glioma patients with concurrent systemic malignancies. One possible explanation is the activation of shared inflammatory and immune pathways that promote tumor progression and impair systemic immune surveillance. Chronic inflammation, cytokine release, and immune dysregulation associated with systemic cancer may create a potential pro-tumorigenic environment, potentially facilitating glioma growth or potentially reducing treatment response [41,42]. Additionally, competing mortality risks from the systemic malignancy and treatment limitations due to overlapping toxicities or decreased tolerance for aggressive therapies may further compromise outcomes [16,18]. The combined physiological burden of two active cancers can also lead to reduced performance status and limited therapeutic options, emphasizing the need for a multidisciplinary and individualized management approach in this complex patient population.

The potential genetic alterations in patients with multiple tumors also warrant further investigation. The presence of a solid systemic malignancy may be indicative of a broader genetic predisposition that influences the development of multiple tumors, as suggested by previous studies investigating patients with multiple primary malignancies [27]. Alterations in genes involved in tumor suppression, DNA repair, or cell cycle regulation might play a role in these patients. Understanding these genetic factors could lead to more tailored treatment strategies and help identify patients at higher risk for developing a second malignancy. Given the complexity of managing dual tumor burden, further studies are needed to explore the genetic alterations in these patients and their potential implications for prognosis and treatment. Identifying specific mutations or pathways involved could open new avenues for personalized therapies that address the unique needs of patients with dual tumors [43,44,45,46,47].

According to our data, treatment in glioma patients should not be reduced solely because of a concurrent systemic malignancy. Instead, therapeutic decisions should be discussed in an interdisciplinary framework and should carefully consider the stage and activity of the systemic disease, the patient’s performance status, prior treatments, and individual preferences to ensure a balanced and personalized approach to care.

This study has several limitations. Firstly, the heterogeneity of systemic malignancies complicates drawing definitive conclusions about their impact on glioma prognosis. Additionally, the lack of comprehensive genetic profiling for all patients may leave some confounding variables unaccounted for. Although the presence of a systemic tumor was identified as an independent prognostic factor, causality cannot be inferred, as potential confounding by age, frailty, and treatment selection may have influenced the observed association. Finally, since this study was conducted at a single institution, the results may not fully reflect broader patient populations. Larger, multicenter studies with detailed genetic analysis are needed to further validate our findings.

In summary, this study provides new insights into the prognostic impact of double tumor burden in glioma patients, addressing a previously underexplored clinical scenario. The large, well-characterized cohort and detailed analysis of both oncological and molecular parameters strengthen the reliability of our findings. However, the retrospective and single-center design, the heterogeneity of systemic malignancies, and the limited sample size of patients with dual tumors must be acknowledged as limitations. Despite these constraints, this study highlights an important and underestimated clinical issue, laying the groundwork for future prospective and molecular studies to validate and expand upon these results.

## 5. Conclusions

Our study demonstrates that the presence of a solid systemic malignancy is an independent prognostic factor for worse overall survival in glioma patients, particularly when the systemic tumor is active. However, when the solid systemic malignancy is in complete remission, no significant difference in outcomes was observed. Further research, including genetic profiling and larger multicenter studies, is needed to better understand the underlying mechanisms and improve treatment strategies for patients with dual tumor burden.

## Figures and Tables

**Table 1:** Differences in baseline characteristics between the two patient cohorts.

	Glioma Only	Glioma Plus Second Tumor	*p*-Value
Median age (IQR)	63 (52–74)	71 (63–79)	**<0.001**
Median preoperative KPS (IQR)	90 (80–100)	80 (70–90)	**0.010**
Median preoperative CFS (IQR)	3 (2–4)	4 (3–5)	**0.011**
IDH mutation	18.3%	7.8%	**0.024**
Median Ki67 expression (IQR)	20 (9–31)	25.3 (11.8–38.8)	**0.045**
MGMT methylation	52.9%	47.3%	0.266
Glioma resection	82.6%	79.7%	0.090
Glioma biopsy	17.4%	21.3%	0.090
Adjuvant therapy for glioma	72.8%	69.3%	0.320
Best supportive care after glioma surgery	8.1%	18.8%	**0.013**
Median KPS at first FU (IQR)	90 (80–100)	80 (70–90)	**<0.001**
Median CFS at first FU (IQR)	3 (2–4)	4 (3–5)	**<0.001**

## Data Availability

The data supporting this study are available from the corresponding author upon reasonable request.
